# Adventure tourism and schistosomiasis: serology and clinical findings in a group of Danish students after white-water rafting in Uganda

**DOI:** 10.1099/jmmcr.0.005141

**Published:** 2018-02-02

**Authors:** Dennis Röser, Stephanie Bjerrum, Marie Helleberg, Henrik Vedel Nielsen, Kim Peter David, Søren Thybo, Christen Rune Stensvold

**Affiliations:** ^1^​ Laboratory of Parasitology, Department of Bacteria, Parasites and Fungi, Statens Serum Institut, Artillerivej 5, DK–2300 Copenhagen S, Denmark; ^2^​ Department of Pediatrics, Herlev Hospital, Herlev Ringvej 75, DK–2730 Herlev, Denmark; ^3^​ Department of Infectious Diseases, Copenhagen University Hospital, Rigshospitalet, Blegdamsvej 9, DK–2100 Copenhagen Ø, Denmark; ^4^​ Department of Infectious Diseases, Hvidovre Hospital, Kettegård Alle 30, DK–2650 Hvidovre, Denmark

**Keywords:** *Schistosoma*, schistosomiasis, tropical diseases, serology, travel, diagnosis

## Abstract

**Introduction:**

Diagnosis of schistosomiasis in travellers is a clinical challenge, since cases may present with no symptoms or a few non-specific symptoms. Here, we report on the laboratory and clinical findings in Danish travellers exposed to *Schistosoma*-infested water during white-water rafting on the Ugandan part of the upper Nile River in July 2009.

**Case presentation:**

Forty travellers were offered screening for *Schistosoma*-specific antibodies. Serological tests were performed 6–65 weeks after exposure. A self-reporting questionnaire was used to collect information on travel activity and health history, fresh water exposure, and symptoms. Seropositive cases were referred to hospitals where clinical and biochemical data were collected. *Schistosoma*-specific antibodies were detected in 13/35 (37 %) exposed participants, with 4/13 (31 %) seroconverting later than 2 months following exposure. Four of thirteen (31 %) cases reported ≥3 symptoms compatible with schistosomiasis, with a mean onset of 41 days following exposure. No *Schistosoma* eggs were detected in stool or urine in any of the cases. Peripheral eosinophilia (>0.45×10^9^ cells l^−1^) was seen in 4/13 cases, while IgE levels were normal in all cases.

**Conclusion:**

Schistosomiasis in travellers is not necessarily associated with specific signs or symptoms, eosinophilia, raised IgE levels, or detection of eggs. The only prognostic factor for infection was exposure to freshwater in a *Schistosoma*-endemic area. Seroconversion may occur later than 2 months after exposure and therefore – in the absence of other diagnostic evidence – serology testing should be performed up to at least 2–3 months following exposure to be able to rule out schistosomiasis.

## Introduction

Schistosomiasis is caused by infection with species of the blood-fluke *Schistosoma*, a parasite transmitted by freshwater snails in mainly tropical and sub-tropical regions. Infection usually occurs in still or stagnant water by cercarial invasion through the skin, the necessary exposure time being as brief as 1–5 min [1, 2]. The first sign of infection may be pruritus, with symptom onset during or shortly after exposure [3], or cercarial dermatitis – an urticarial or erythropapulous rash – developing within hours after exposure and lasting for a few days. Schistosomiasis typically develops within 14–84 days after exposure, when migrating schistosomules and *Schistosoma* eggs can cause a systemic hypersensitivity reaction, resulting in fever, and pulmonary and abdominal symptoms [4]. The disease may progress into chronic schistosomiasis, a fibrosis-obstructive disease, where the host immune response towards *Schistosoma* eggs involves granulomatous reactions in affected organs, with a risk of malignant transformation [1, 5]. Chronic schistosomiasis can present with symptoms months or years after exposure, with haematuria or gastrointestinal discomfort as the most common presentations. Though uncommon, neurological and cardiac symptoms may also develop, with potentially severe consequences [6].

Diagnosing schistosomiasis in travellers returning from endemic areas is a well-known clinical challenge. Microscopy for eggs in patient samples has very low diagnostic sensitivity in low-burden infections [7], whereas serology has higher sensitivity and specificity in cases with no previous exposure to *Schistosoma* [4]. However, serology cannot identify *Schistosoma* at the species level [6] or be used to monitor treatment efficacy [8, 9], and since the efficacy of standard treatment using praziquantel varies and is dependent on the host immune response [10], asymptomatic individuals with recent relevant exposure and lacking detectable ova during the initial screening phase pose a challenge in terms of clinical control. Previous studies of *Schistosoma*-naïve travellers with relevant exposure have seen high attack rates of schistosomiasis [11, 12]. In this study, we describe a cluster of seropositive cases among 40 Danish students and teachers participating in white-water rafting on the Ugandan part of the upper Nile River in July 2009.

## Case report

After returning to Denmark – and 6 weeks after exposure to the *Schistosoma*-infested water – one of the students (the index case) sought medical attention for haematuria and tested positive for *Schistosoma*-specific antibodies. This prompted the physician who had advised the group on travel health precautions prior to departure (including malaria prophylaxis) to recommend screening of the entire group for *Schistosoma*-specific antibodies. We identified the members of the group through this physician. Questionnaires were sent to the participants in order to collect data on travel history (including travel activity in regions to which schistosomiasis is endemic), individual health history (including previous schistosomiasis), fresh-water exposure during travelling (including river-rafting events), and symptoms experienced during travelling and after returning to Denmark.

Seropositive cases were referred to the Department of Infectious Diseases at either Hvidovre University Hospital (Hvidovre, Denmark) or Copenhagen University Hospital, Rigshospitalet (Copenhagen, Denmark), from where we collected clinical and paraclinical data. We used a standardized chart to record signs and symptoms of schistosomiasis (e.g. fever, cough, sweating, myalgia, headache, urticaria, abdominal complaints, blood in urine or stool, hepatosplenomegaly and lymphadenopathy), laboratory results and treatment received. We recorded any presence of dysuria and tested for haematuria. In addition, we repeated *Schistosoma-*specific serology when possible and added additional diagnostic testing, including tests for IgE antibody levels and eosinophilia (eosinophilia was defined as an eosinophil count of >0.45×10^9^ cells l^−1^), screening for blood, leukocytes and protein in the urine, and microscopy of one faecal sample for ova and parasites. Twenty-four-hour urine samples were collected and examined by microscopy for eggs of *Schistosoma*, using an in-house ninhydrin sedimentation–filtration assay. All tested individuals had their first serological screening performed 6–10 weeks after exposure, and serological testing was repeated up until 65 weeks following exposure.

The study group consisted of 40 participants, and the questionnaire response rate was 39/40 (98 %). In total, 35/39 had been exposed to freshwater either on a one-day rafting trip (30/35) or over two days of rafting and occasional swimming in the Upper Nile (5/35). All participants were in good health prior to departure, with no known previous exposure to *Schistosoma*-infested water.

**Fig. 1.**
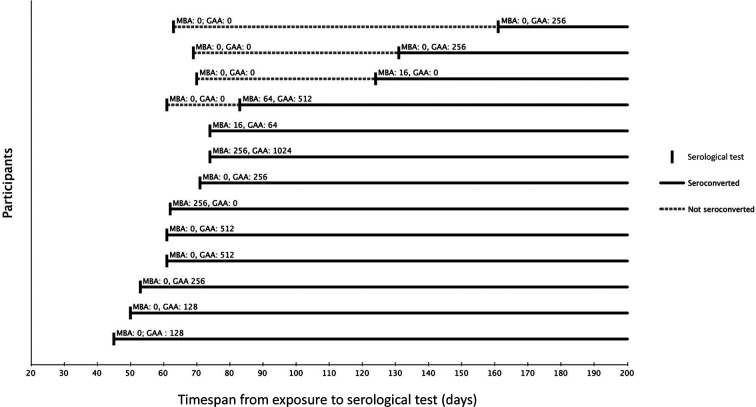
Overview of the time of seroconversion in the 13 individuals developing antibodies against *Schistosoma*: timespan from exposure to serological test in days. Reciprocal MBA and GAA antibody titres are displayed. Four participants had more than one test performed.

A total of 13/35 (37 %) of the exposed participants were found to be seropositive for *Schistosoma*-specific antibodies, 12 (92 %) of whom developed antibodies against *Schistosoma* gut-associated antigen (GAA; see below). In 9/13 individuals, seroconversion was evident on the first serology test taken 45 to 74 days (median 61 days) following exposure (Table 1, Fig. 1). For the remaining four individuals, seroconversion occurred later than 2.5 months following exposure, as they tested negative on the first test taken 61–70 days (median 66 days) after exposure, but positive on the second test taken 83–161 days (median 128 days) after exposure (Table 1, Fig. 1).

**Table 1.** Serology profiles of the 13 individuals who seroconverted Positive serology results are shown in bold.

		**1st serology test**			**2nd serology test**			**3rd serology test**			**4th serology test**		
Gender	Study no.	No. of days since exposure	MBA	GAA	No. of days since exposure	MBA	GAA	No. of days since exposure	MBA	GAA	No. of days since exposure	MBA	GAA
F	4	70	0	0	124	**16**	0	452	**32**	**16**			
F	8	74	**16**	**64**	134	0	**256**	333	0	**128**			
F	9	69	0	0	131	0	**256**	413	0	**32**			
F	10	45	0	**128**	114	0	**512**						
F	11	63	0	0	161	0	**256**						
F	15	53	0	**256**	61	0	**2048**	268	0	**256**			
M	21	61	0	0	83	**64**	**512**	102	**32**	**128**	322	0	**64**
M	25	50	0	**128**	73	0	**256**	267	0	**512**			
M	28	62	**256**	0	413	**64**	0						
M	30	71	0	**256**	106	0	**512**						
M	31	61	0	**512**	81	0	**1024**	426	0	**256**			
M	32	61	0	**512**	124	0	**512**	183	0	**256**	269	0	**128**
M	36	74	**256**	**1024**	104	**64**	**1024**	284	**128**	**128**			

F, Female; M, male.

Based on questionnaire data, 4/13 seropositive participants had experienced ≥3 symptoms compatible with schistosomiasis, with a mean onset of 41 days following the last possible exposure. The symptoms were unspecific and included fever, stomach pain, nausea, diarrhoea, headache and rash experienced in different combinations. One case (the index case) reported complaints of macroscopic haematuria. By clinical examination, 3/13 seropositive participants had palpable lymph nodes and 1 individual had abdominal tenderness.

Faecal and urine tests were offered to all participants. Faecal samples were obtained from 4/13 seropositive participants and 24 h urine samples from 8/13 participants collected 12–16 weeks after exposure. The remaining seropositive individuals failed to submit faecal or urine samples for testing. No eggs were detected by microscopy in any of the faecal or urine samples. Urine samples were tested for 9/13 participants, with all testing negative for blood, leucocytes and protein. Peripheral eosinophilia was detected in 4/13 seropositive participants (mean concentration 0.8×10^9^ cells l^−1^), with 1 individual exhibiting persistent eosinophilia for more than 6 months following exposure, despite treatment. Other standard biochemical analysis revealed no other abnormalities.

Time from exposure to clinical examination and repeated serology at the referral site varied. The mean time from exposure to first clinical consultation at hospital was 5 months.

## Investigations

Serological analyses were performed at the Statens Serum Institut, Copenhagen, Denmark. Each participant was tested for *Schistosoma*-specific antibodies in ≥2 serum samples, with ≥1 sample collected later than 3 months after exposure. We used an immunofluorescence antibody test (IFAT) for GAA and membrane-bound antigen (MBA) from *Schistosoma mansoni* larvae [13]. Sera were diluted twofold, starting at a dilution of 1 : 16 up to 1 : 256, as described elsewhere [13]. A GAA and/or MBA titre ≥16 was defined as positive, as recommended [13]. GAA is the more reliable marker of acute schistosomiasis in individuals from non-endemic areas, as MBA is less specific, and so interpretation should reflect individual exposure [13].

After completion of the study, serum samples were sent to Leiden University Hospital, Leiden, The Netherlands, for confirmatory serology (IFAT for GAA, ELISA for *Schistosoma* egg antigen antibodies). Confirmatory serology test results from Leiden University Hospital showed 96 % concordance with the Statens Serum Institut results for GAA (data not shown).

Freshwater exposure was defined as either low (<5 water dips) or high (>5 water dips), and both the number of times swimming in river (slower moving waters) or falling off the boat in rapids (turbulent waters) were recorded. The data collection period was defined as the time from exposure to 1 year after referral, and in case of multiple exposures, time of exposure was identified as the last possible time of exposure. Data collection ended when each participant had submitted sera for testing ≥2 times with ≥1 sample collected later than 3 months following exposure. Statistical analysis was performed using SAS 9.2 for Windows.

## Treatment

None of the 13 seropositive travellers were treated with praziquantel prior to the first clinical consultation at hospital. Patients in a total of 12/13 of the seroconverted cases were prescribed standard treatment with praziquantel (40 mg/kg body weight given as a single dose); the last case failed to make a clinical appointment despite several phone calls and letters.

## Outcome and follow-up

All who were seen by an infectious disease specialist were offered clinical evaluation after 6 months, including analysis of blood and urine samples. Several had blood and urine tests performed in a different clinical setting, typically one closer to home.

Five of the thirteen seropositive individuals answered the question ‘how long did symptoms last’, and they answered ‘for 2–4 days’. It might be safe to say that those who did not respond to this question did not experience any symptoms. Seronegative participants were not referred for clinical examination, but were offered follow-up serology by their family physicians.

## Discussion

In the present study, we detected *Schistosoma*-specific antibodies in more than one third of the Danish individuals participating in a white-water rafting trip on the Ugandan part of the Upper Nile in 2009. Morgan *et al*. [14] observed seroconversion in 17 % of participants in a kayaking event on the river Upper Nile in Uganda. This study further noted that 20 % of participants used praziquantel as a preventive measure immediately after exposure [14], despite the fact that praziquantel as a preventative measure is not recommended as it only affects adult worms, not larvae. Jauréguiberry *et al*. summarized 13 reports on clusters of schistosomiasis, reporting attack rates from 65 to 100 %; 2/3 of the clusters originated from sub-Saharan Africa, the remaining 1/3 from Brazil [6]. One study reported clusters of schistosomiasis in 3/20 rafting trips on the river Omo in Ethiopia, with a seroprevalence ranging from 59 to 90 % in affected groups [15].

The length of exposure to cercariae is known to influence the risk of contracting infection with *Schistosoma* [16, 17]. In the present study, 13/35 (37 %) travellers developed *Schistosoma*-specific antibodies subsequent to 1 to 2 days of river rafting on the Nile River near Jinja, Uganda. In comparison, the river rafting cohorts described by Swartz *et al*. [15] saw higher attack rates (59–90 %) after 8–15 days of exposure on the river Omo in Ethiopia, and Morgan *et al*. [14] saw a lower attack rate (17 %) after 7 days of exposure on the Nile River, also near Jinja, Uganda. As such, the recorded length of exposure expressed as ‘days’ did not offer a complete explanation for the observed differences in attack rate between the three studies. As environmental and seasonal factors are known to influence the risk of becoming infected [17], we investigated whether there were any differences in rainfall between our cohort and that of Morgan *et al*., as they were both exposed in the same geographical location (Jinja, Uganda); however, we found that the level of rainfall in the two periods (July 2009 and November 2007) was somewhat similar [18]. We, therefore, attribute the observed differences to other factors, possibly differences in cohort behaviour and activities, as the cohort described by Morgan *et al*. [14] comprised not only river rafters, but also spectators, swimmers and kayakers.

When using IFAT for detection of *Schistosoma*-specific antibodies in individuals from non-endemic regions, GAA is considered more specific and is preferred over MBA, which in turn is appropriate for detection of chronic infection in patients from endemic regions [13]. In accordance with this, we found a low and transient MBA titre in the absence of a positive GAA result in three cases and interpreted these results as negative (data not shown); however, we also observed one case where GAA-specific antibodies appeared only after the emergence of MBA-specific antibodies, and one case for whom a consistently high MBA titre was detected, but who failed to develop GAA antibodies (Table 1, Fig. 1). For this reason, we suggest that a patient presenting with a negative GAA result and a low MBA titre should be re-tested, especially if the sampling time is close to the time of exposure, since four cases in this study seroconverted only 2 months after exposure or later. Late seroconversion is consistent with observations by Grandiére-Pérez *et al*. [19], who found a mean interval from exposure to seroconversion of 46 days (range 27–100 days). In the present cohort, performing serology 3 months after exposure would have detected all 13 cases, but we cannot rule out that a systematic 6-month-post-exposure serology would have detected cases that we considered negative.

Infection with schistosomiasis prior to exposure might also result in positive serology and cannot be ruled out, since several participants had visited regions endemic to schistosomiasis previously. We do consider this unlikely, as no one in the cohort had any known previous infections, and none had visited sub-Saharan Africa, which is the principal region of exposure to *Schistosoma* for Danish travellers [20]. Nevertheless, exposure to *Trichobilharzia –* a parasite of wide geographical distribution including Europe *–* might induce low and/or transient anti-MBA antibody titres.

In the present study, seroconversion was not statistically significantly associated with levels of self-reported exposure (e.g. number of dips in rapid and stagnant waters; data not shown). Clinical identification of cases by symptoms alone was not possible. Moreover, no ova were detected by microscopy, which is not surprising given the limited sensitivity of microscopy of stool and urine for schistosomiasis in travellers from non-endemic countries, who usually have a very low parasite burden; other studies have shown microscopy of rectal biopsies to have a higher sensitivity than microscopy of stool concentrates, but in this study, no rectal biopsies were available for analysis. Peripheral eosinophilia was found in only 50 % of seropositive cases, indicative of helminth infection.

Based on the geographical setting, we consider *S. mansoni* the more likely species to be the cause of infection in this cohort; however, since no eggs were identified, and since no molecular analyses were performed on genomic DNA from stool or urine, this cannot be corroborated.

This study shows that even brief exposure to *Schistosoma*-infested waters carries a high risk of infection, and seroconversion occurs sometimes later than 2 months after exposure. In patients with few symptoms and no detectable ova, seropositivity becomes the sole indicator of infection. In this study, no ova were detected, and more than half of the cases presented no or few symptoms of schistosomiasis. Similar observations have been reported before [9, 21], and considering the longevity of a *Schistosoma* infection, we recommend that seropositive patients be treated in all cases, even if all other clinical indicators are absent. Control of treatment efficacy will still present a problem though, and physicians should mind the prepatency time and ensure that antibody tests are negative up to at least 3 months from exposure to be able to rule out a diagnosis of schistosomiasis.

To our knowledge, no information was provided by the travel company or the school pre-travel on schistosomiasis to educate and raise awareness prior to the start of the trip. Efforts are being taken to put measurements in place to improve travel-related health protection in Danish tourists. Such measurements have recently been implemented in Great Britain [22, 23].
